# The Effect of Bovine Parathyroid Hormone Withdrawal on MC3T3-E1 Cell Proliferation and Phosphorus Metabolism

**DOI:** 10.1371/journal.pone.0120402

**Published:** 2015-03-16

**Authors:** Shuangxin Liu, Weiping Zhu, Sijia Li, Tongxia Cui, Zhonghe Li, Bin Zhang, Zhuo Li, Jianxiong Wu, Xinling Liang, Zheng Lin, Wei Shi

**Affiliations:** 1 Department of Nephrology, Guangdong General Hospital, Guangdong Academy of Medical Sciences, 106 Zhongshan No. 2 Road, Guangzhou, China; 2 Department of Nephrology, the Fifth Affiliated Hospital of Sun Yat-sen University, No.52 Meihua Road, Zhuhai, China; University of Massachusetts Medical, UNITED STATES

## Abstract

Hypocalcemia and hypophosphatemia are common complications after parathyroidectomy (PTX). Sudden removal of high circulating levels of parathyroid hormone (PTH) causes decreased osteoclastic resorption resulting in a decreased bone remodeling space. These phenomena are likely due to an increased influx of calcium and phosphorus into bone. However, there are currently no data to support this hypothesis. In this study, we found that PTX significantly reduced levels of PTH, calcium and phosphate. Compared with preoperative levels, after 1 year, postoperative PTH, calcium and phosphate levels were 295.6 ± 173.7 pg/mL (P < 0.05), 86.62 ± 15.98 mg/dL (P < 0.05) and 5.56 ± 2.03 mg/dL (P < 0.05), respectively. We investigated continuous bovine PTH administration as well as withdrawal of bovine PTH stimulation in the mouse osteoblast precursor cell line MC3T3-E1. MC3T3-E1 cells were cultured with continuous bovine PTH treatment for 20 days or with transient bovine PTH treatment for 10 days. High doses of continuous bovine PTH exposure strongly reduced cell proliferation, alkaline phosphatase activity and the number of mineralized calcium nodules. However, withdrawal of bovine PTH (100 ng/mL) significantly increased the number of mineralized calcium nodules and caused a rapid decline in calcium and phosphorus content of culture medium. In conclusion, continuous exposure to bovine PTH inhibited osteoblast differentiation and reduced the formation of mineralized nodules. However, this inhibition was removed and mineralized nodule formation resumed with withdrawal of bovine PTH. According to the results of our clinical examinations and *in vitro* experiments, we hypothesize that the sudden removal of high levels of PTH may cause an increased influx of calcium and phosphorus into bone after PTX.

## Introduction

Since parathyroid hormone analogs become the only US FDA-approved anabolic drug, intermittent parathyroid hormone (iPTH) has been widely used in clinic to treat bone loss due to conditions such as osteoporosis that increases bone formation by osteoblasts.[1] PTH increases osteoblastogenesis and osteoblast maturation, promotes osteoblast survival and inhibits osteoblast apoptosis, thereby increasing osteoblast number and function.[2,3] A low dose and intermittently PTH seems to be able to exert positive effects on bone volume and microarchitecture.[4] PTH signals via its receptor on the osteoblast membrane, and osteoclasts are impacted indirectly via the products of osteoblasts.[5] Binding of PTH to PTH receptor 1 also activates the canonical Wnt signaling pathway, therefore increasing osteoblastic differentiation and bone formation.[6] The anabolic effects of iPTH are likely due to a variety of different mechanisms, including induction of immediate-early genes, increased expression and/or activity of essential osteoblast transcription factors, and decreases sclerostin levels, thereby removing inhibition of Wnt signalling which is required for PTH's anabolic actions.[7,8,9]

Bone mass and turnover are maintained by the coordinated balance between bone formation by osteoblasts and bone resorption by osteoclasts, under the control of many cytokines.[10,11] Osteoclasts are multinucleated cells which arise from fusion of bone marrow monocytes and macrophages that are specialized to carry out bone resorption, whereas osteoblasts are responsible for osteogenesis.[12] Increased osteoclast number and activity is closely related to a variety of bone loss conditions, resulting in pathological fracture and seriously affecting the patient's quality of life.[13] In chronic kidney disease (CKD) patients with secondary hyperparathyroidism (SHPT), continuous elevation of parathyroid hormone (PTH) increases osteoclast activity, stimulates bone resorption, mobilizes bone calcium, and disrupts the balance between osteoclasts and osteoblasts.

The consequent degradation of the inorganic bone matrix by activated osteoclasts causes calcium and phosphorus to be released from dissolved hydroxyapatite crystals.[14] SHPT contributes to the hyperphosphatemia and hypercalcemia that commonly affect patients on dialysis. Parathyroidectomy (PTX) is recommended as an effective treatment for severe SHPT with hyperphosphatemia and hypercalcemia. PTX causes a rapid decrease in serum calcium and phosphorus levels after successful removal of hyperactive parathyroid gland(s). The resulting hypocalcaemia and hypophosphatemia is believed to be due to the greatly increased skeletal usage of calcium and phosphorus, thought to occur as a result of removal of the effect of high circulating PTH levels on bone, with immediate arrest of bone resorption in the face of continuing and enhanced bone formation. However, there is insufficient evidence to support this. To address this, we investigated the changes of serum calcium and phosphorus after PTX in SHPT patients. In order to clarify the mechanism of hypocalcaemia and hypophosphatemia in these patients, we examined the impact of PTH of the osteoblastic cell line MC3T3-E1.

## Materials and Methods

### 1. Patient selection

This study was approved by the ethics committee of Guangdong General Hospital (Guangdong, China). Written informed consent for the study was obtained from all participants. All dialysis patients with SHPT who presented to Guangdong General Hospital for PTX from May 2010 to June 2013 were eligible for enrollment into the study. Patients were enrolled and underwent preoperative and postoperative examination for evaluation of calcium and phosphorus with a planned follow-up of 12 months for repeat laboratory testing. Because of postoperative hypocalcemia, intravenous calcium and oral calcium supplementation were required in all patients. In addition, calcitriol was given to all patients who underwent initial PTX (0.25–0.5μg /d, depending on serum calcium level). In all dialysis patients a high calcium dialysate was used.

### 2. Reagents

MC3T3-El cells (subclone 14) were obtained from ATCC (Manassas, VA). Dulbecco’s Modified Eagle’s Medium (DMEM) and penicillin/streptomycin (10,000 IU/mL and 10 mg/mL) were purchased from Gibco Inc. (Grand Island, NY). Fetal bovine serum (FBS) was obtained from Biological Industries (Kibbutz Beit Haemek, Israel). Bovine PTH (1–34) was acquired from Bachem Bioscience Inc. (King of Prussia, PA). AKP staining kits were purchased from Jiancheng Bioengineering Institute (Jiancheng, Nanjing, China). MTT kits were acquired from Beyotime Institute of Biotechnology (Beyotime, Nanjing, China). β-Glycerophosphate and L-ascorbic acid 2-phosphate were purchased from Sigma-Aldrich (St. Louis, MO).

### 3. Administration of bovine PTH (bPTH)

Bovine PTH (1–34) powder was dissolved in a solution of 0.1% bovine serum albumin (BSA) to create a reconstituted stock solution that could be stored at −20°C. Control cultures were treated with the same concentration of BSA in order to exclude any possible effects of the vehicle. For continuous exposure, bPTH was present in the culture medium for the entire 20-day culture period, with fresh bPTH added with each medium change. The final concentrations of bPTH in the culture medium were 100, 10, 1, or 0.1 ng/mL. For transient exposure, bPTH (100 ng/mL) was added to the culture medium from day 1 to day 10, but not from days 11 to 20.

### 4. Cell culture

MC3T3-E1 cells were cultured in 25 cm^2^ plastic flasks containing α-MEM supplemented with 10% (v/v) FCS, 100 U/mL penicillin, 100 μg/mL streptomycin, and 2 mM glutamine. Cells were incubated at 37°C in an atmosphere of 95% air and 5% carbon dioxide until they reached 90% confluence, then harvested in trypsin/EDTA, counted using a hemocytometer, re-seeded at 10^4^ cells/cm^2^, and allowed to adhere overnight prior to treatment.

### 5. Methyl thiazolyl tetrazolium (MTT)-dye assays

MC3T3-E1 cells were seeded at 1 × 10^3^ cells/well in 96-well culture plates and cultured for 2 to 20 days in the presence of bPTH. Plates were evaluated every 2 days by MTT assay. Cell culture medium was removed using a needle and syringe. Cells were incubated with 10 μL of MTT solution at 37°C for 4 hours. DMSO (200 μL) was added to each well and mixed thoroughly to ensure that all crystals were dissolved. Plates were incubated at 37°C for 4 hours. Absorbances were read using a microplate reader at a wavelength of 595 nm. Cell proliferation rate = (Value of the experimental group—Value of the control group) / (Value of the control group) × 100%.

### 6. AKP Staining

MC3T3-E1 cells were cultured in 10 cm plastic plates. After 20 days in culture, cells were rinsed with PBS, fixed in 4% paraformaldehyde, rinsed with distilled water, and overlaid with 1.5 mL of 0.2 M 2-amino-2-methyl-1, 3-propanediol solution plus 1 mg/mL naphthol AS-BI phosphate disodium salt in 0.1 M Tris-HCl (pH 9.75) and 0.01 M NaOH. Cells were incubated at 37°C for 30 minutes in the dark, then rinsed with distilled water and counterstained with hematoxylin. Plates were washed with distilled water and observed by microscopy.

### 7. AKP activity assays

After 4 to 20 days in culture, osteoblasts in 6-well plates were washed with PBS and lysed using a probe sonicator for 20 s in 0.1 M Tris buffer (pH 7.2) containing 0.1% Triton X-100 at 4°C. Sonicated cell lysates were centrifuged for 10 min at 1500 × *g* at 4°C, and supernatants were used for the assays. AKP activity was assayed using AKP assay kits at 37°C. In brief, cell extracts (50 μL) were aliquoted into 96-well plates together with 100 μL of buffer-substrate solution containing 0.5% potassium ferricyanide, 40 mM ρ-nitrophenyl phosphate disodium, and 0.1 M Tris-HCl (pH 10). After incubation for 15 min, 150 μL of 0.3% 4-amino-antipyrine was added to each well and mixed gently. Absorbances were measured using a microplate reader at a wavelength of 490 nm. A standard curve was prepared with ρ-nitrophenol. Each value was normalized to the protein concentration and expressed as U/g per l.

### 8. Mineralization assays

The mineralization of MC3T3-E1 cells was determined using Alizarin Red S staining as described previously.[15] Confluent cells were grown for 20 days in DMEM supplemented with 10% FBS, 100 U/mL penicillin, 100 μg/mL streptomycin, 2 mM glutamine, 25 μg/mL ascorbic acid 2-phosphate, 0.1 μM dexamethasone, and 10 mM β-glycerophosphate; culture medium was replaced every 48 hours. Cells were fixed with ice-cold 70% ethanol and stained with 40 mM Alizarin Red S (Sigma) to detect calcification.

### 9. Determination of calcium and phosphorus content

MC3T3-E1 cell culture medium was collected at the end of a 48-hour period during the mineralization assays. Calcium and phosphorus contents were determined using inductively coupled plasma atomic emission spectrophotometry (ICP-AES; model: IRIS Advantage (HR), Thermo Jarrel Ash Corporation, Boston, MA) after wet digestion with nitric acid and perchloric acid.

### 10. Statistical analysis

Results are expressed as the mean ± standard error of the mean (SEM). Differences were analyzed using t-tests performed using SPSS 13.0 software (SPSS, Chicago, IL). All statistical tests were two-sided, and *P*-values less than 0.05 were considered statistically significant.

## Results

### 1. Patient characteristics

Demographics and pertinent patient characteristics are presented in Table 1. Twenty-five patients were enrolled and underwent PTX, with preoperative and postoperative examination for evaluation of calcium, phosphorus and PTH and a follow-up period of 12 months. As indicated in Table 2, PTX significantly reduced PTH, calcium and phosphate. Compared with preoperative values, by 1 year post-operatively PTH, calcium and phosphate levels were 295.6 ± 173.7 pg/mL (*P* < 0.05), 86.62 ± 15.98 mg/dL (*P* < 0.05) and 5.56 ± 2.03 mg/dL (*P* < 0.05), respectively.

**Table 1:** Baseline characteristics of patients with parathyroidectomy.

Characteristic	Value
Age (year) Mean (±SD)	50.1 ± 18.4
Gender: male/female	11/14
Diabetes	8.0% (2)
Calciphylaxis	4.0% (1)
Duration of dialysis (year)Mean (±SD)	9.4 ± 12.6
Calcium Carbonate	80.0% (20)
Hypertension	76% (19)
Mean BP, systolic (range) (mmHg)	115 (85–170)
Mean BP, diastolic (range) (mmHg)	65 (51–120)

**Table 2:** Preoperative and postoperative biochemical values for dialysis patients following parathyroidectomy.

	Preoperative(n = 25)	Postoperative (n = 25)		
		3 hours	1 week	1 year
Intact parathyroid hormone (pg/mL)	1573.1 ± 699.4	106.3 ± 86.7*	238.5 ± 151.4*	295.6 ± 173.7*
serum calcium (mg/dL)	98.08 ± 7.54	80.36 ± 14.17*	72.70 ± 12.52*	86.62 ± 15.98*
Serum phosphorus (mg/dL)	8.03 ± 1.86	4.37 ± 1.52*	3.70 ± 1.27*	5.56 ± 2.03*

Values are mean ± SEM. Numbers in parentheses refer to the number of patients for whom data were available. Compared with preoperative, postoperative PTH, calcium and phosphate levels were considered statistically significant.

* *P* < 0.05.

### 2. Effects of PTH withdrawal on osteoblast proliferation

**Fig 1:**
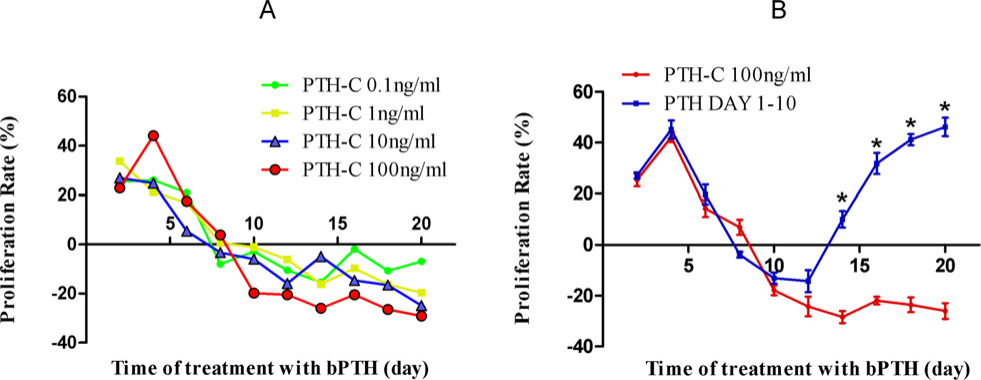
Effects of PTH withdrawal on osteoblast proliferation. Cells were grown in microtiter plates in a final volume of 200 μL culture medium per well for 2–20 days. MTT assays were performed after 48 h. (A) Cell proliferation appears to be downregulated in the first 8 days, and inhibited after 10 days of culture in the continuously-treated bPTH groups. The effect of inhibition increases with the concentration of bPTH. (B) Although osteoblast proliferation coordinately declined in the PTH-C 100 ng/mL and PTH Day 1–10 groups, it rebounded after bPTH withdrawal in the PTH Day 1–10 group. *Significantly different compared to the PTH-C 100 ng/mL group at the same time point, *P* < 0.05.

To investigate the effects of PTH on MC3T3-E1 cell proliferation, early passage cultures were separated into six groups. The treated groups (PTH-C) were exposed to different concentrations of PTH (100, 10, 1, or 0.1 ng/mL) for 20 days. Another group (PTH Day 1–10) had bPTH withdrawn after treatment with 100 ng/mL bPTH for 10 days. In the continuously-treated groups, we observed significantly decreased proliferation compared with control cultures (Fig. 1A). However, a rebound of proliferation was observed in the PTH Day 1–10 group after bPTH withdrawal (Fig. 1B). There were significant differences between the PTH Day 1–10 group and the PTH-C 100 ng/mL group at the same time points (days 14, 16, 18, and 20).

### 3. Effects of PTH withdrawal on AKP activity

**Fig 2:**
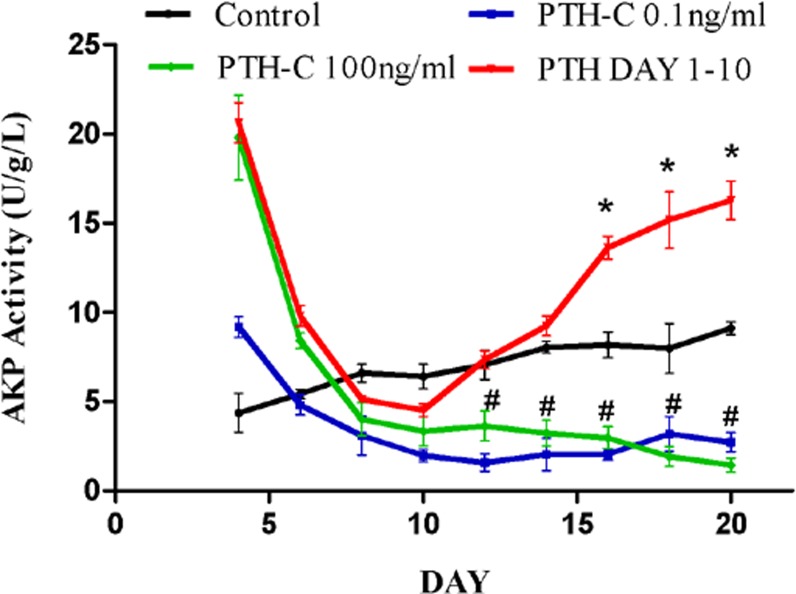
Effects of PTH withdrawal on AKP activity. In control cultures, AKP activity increased with time and reached a plateau after 14 days. In contrast, AKP activity declined from day 4 to day 10 and did not increase over time in the continuous PTH cultures. In the PTH Day 1–10 group that was treated with bPTH on days 1 to 10, AKP activity rebounded after withdrawal of bPTH and was significantly higher than controls and continuously bPTH-treated cells from day 16. AKP activity did not fully recover until day 20. *^, #^Significantly different compared to control at the same time point, *P* < 0.05.

**Fig 3:**
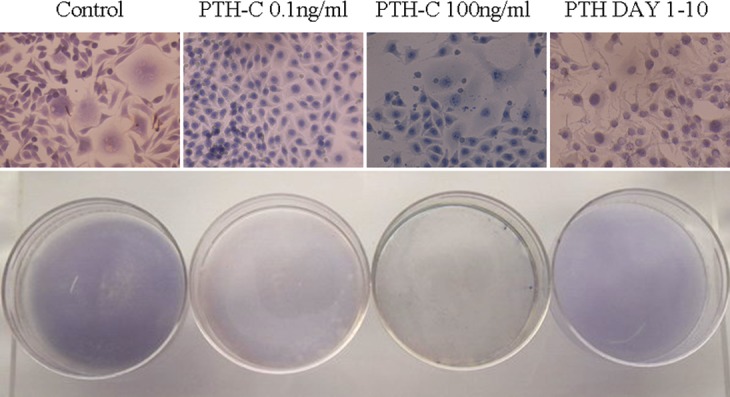
AKP histochemical staining. AKP activity of osteoblasts was evaluated by histochemical staining. AKP granules that are abundantly expressed in the cytoplasm of mature osteoblasts were stained red-brown with purple nuclei. The control group had the highest AKP activity of all groups. AKP granules in the PTH Day 1–10 group exhibited light red staining, while reddish staining was observed in the PTH-C 0.1 ng/mL group, and red-brown staining was observed in controls. AKP activity was weakest in the PTH-C 100 ng/mL group and few AKP granules were formed.

AKP-positive cells were quantified using enzymatic activity assays and histochemical staining. After 20 days in culture, a significant decrease in AKP activity was observed in the continuous exposure groups (PTH-C 100 ng/mL and PTH-C 0.1 ng/mL) compared with controls (Fig. 2). However, AKP activity recovered gradually after bPTH withdrawal in the transient bPTH exposure group. Histochemical staining of osteoblasts demonstrated that the control group had high AKP activity, with red-brown granules apparent in the cytoplasm and purple granules in the nuclei (Fig. 3). Cells in the PTH Day 1–10 group stained light red in the cytoplasm and the nuclei were purple. However, the cytoplasm of cells in the PTH-C 0.1 ng/mL group showed reddish staining. Cytoplasmic AKP staining of the PTH-C 100 ng/mL group was too weak to observe.

### 4. Effects of PTH withdrawal on mineralization

**Fig 4:**
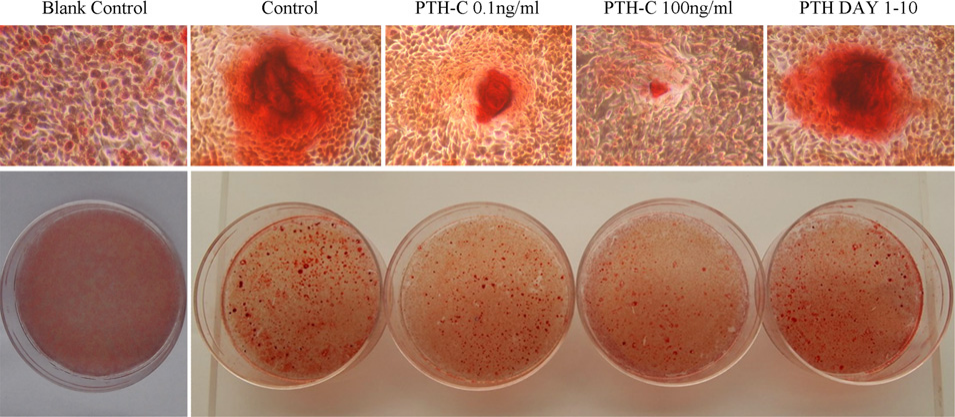
Effects of PTH withdrawal on mineralization. Mineralized nodule formation was assessed by Alizarin red S (ARS) staining 20 days after differentiation. The blank control group without the supplement of bPTH and the osteogenic induction in the cell culture was performed as a negative control. Original magnification 100×.

We evaluated the effects of transient or continuous bPTH exposure on mineralized nodule formation using Alizarin Red S staining. Numerous calcium nodules comprising AKP-positive cells in a mineralized matrix were observed in the control cultures (Fig. 4). bPTH induced different effects on the number of bone nodules depending on the exposure time and concentration of bPTH. There were more calcium nodules in the PTH-C 0.1 ng/mL group compared to the PTH-C 100 ng/mL group, but fewer than in the control group. In the PTH Day 1–10 group, there were fewer calcium nodules compared to controls. PTH withdrawal (100 ng/mL) induced a significant increase in the number of mineralized nodules compared to the continuously-treated group.

### 5. Change of calcium and phosphorus in culture

**Fig 5:**
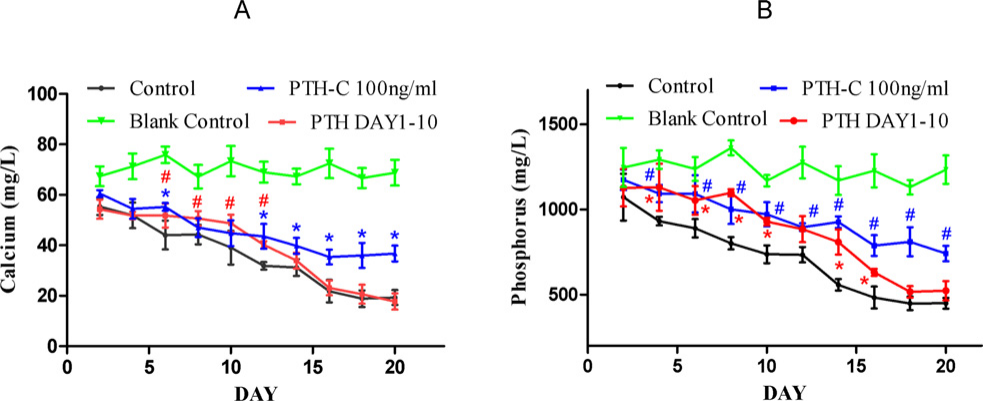
Effects of PTH withdrawal on calcium and phosphorus in culture. Calcium and phosphorus derived from control and bPTH treated cultures at different time points. In the control group, there was a gradual decline of calcium and phosphorus content that reached a plateau after day 16. Similarly, calcium and phosphorus content slowly decreased with time in continuous bPTH cultures. Although the initial calcium and phosphorus content in cultures treated with transient bPTH decreased more slowly than in controls, they showed a rapid decline after bPTH withdrawal on day 10 and attained similar levels as control. *^, #^Significantly different compared with control at the same time point, *P*< 0.05.

To further investigate the effects of PTH withdrawal (100 ng/mL) in each 48-hour incubation cycle, we quantified the calcium and phosphorus content in culture medium (Fig. 5). bPTH exposure induced diverse effects on the calcium and phosphorus content of culture medium depending on the exposure time. The calcium and phosphorus content of culture medium in the PTH-C 100 ng/mL group were higher than in the control group. The calcium and phosphorus content in the PTH Day 1–10 group was also higher than in the control group in the first 10 days, but decreased after bPTH withdrawal at day 10 and returned to control group levels.

## Discussion

Secondary hyperparathyroidism (SHPT) is a common complication of end stage renal disease. SHPT directly contributes to the development of hyperphosphatemia among patients with little or no residual renal function. In this situation, PTH can no longer enhance phosphorus excretion in the urine. The biological action of PTH in enhancing bone resorption when plasma PTH levels are elevated may contribute to hyperphosphatemia by promoting phosphorus release from bone.[16,17] Treatment of SHPT with PTX or calcimimetic agents has been shown to lower PTH levels with concurrent reductions in serum phosphorus.[18,19,20,21]

In our clinical study, biochemical aberrations were observed in 25 dialysis patients following PTX. Unique postoperative electrolyte aberrations were observed 1 week and 1 year after PTX, including severe hypocalcemia and hypophosphatemia (Table 2). This phenomenon is broadly referred to as “hungry bone syndrome” (HBS). HBS occurs in up to 95% of patients with SHPT [20,22,23], and is a general post-PTX complication that can be prevented and treated with oral and intravenous calcium supplementation and/or active vitamin D metabolites.[24,25] Proposed mechanisms for this phenomenon include ongoing phosphorus deposition into bone, phosphorus deposition into soft tissue, diminished intestinal phosphorus absorption, or increased intestinal phosphorus loss.[26] However, there were no changes in the diets of these patients or in the dialysis therapeutic regimens after PTX. Additionally, there is no direct evidence to prove that phosphorus is deposited into soft tissue. In contrast, massive soft tissue calcification resorption was observed in hemodialysis patients after PTX.[27,28] We have also found that phosphorus excretion in the dialysis solution and stool of post-PTX patients is not increased (data not shown). Therefore, we focused on bone as the target organ of phosphorus deposition. After surgery, a rapid shift of calcium from the bloodstream to bone occurs with a marked increase in bone remineralization, which is caused by abrupt removal of elevated PTH levels.[19,20,22] Bone biopsies showed extensive remineralization, implying active deposition of calcium and phosphate into the skeleton.[29] Mineralization of bone remodeling units may lead to hypocalcemia and hypophosphatemia until the new osteons are complete.

Previous studies have revealed that there are a few changes in the bone mineral density (BMD) of hyperparathyroidism patients after PTX.[30,31,32,33,34,35] A historic cohort of 236 primary hyperparathyroidism (PHPT) patients who underwent DXA scans pre-surgery and 1 year after surgery revealed that BMD was improved at both the hip (1.5%) and the spine (2.6%).[31] Furthermore, there were positive associations between preoperative plasma PTH levels and postoperative BMD increases.[31,32] Another observation of 14 patients with severe secondary hyperparathyroidism who underwent iliac bone biopsy before surgery and 1 week after PTX showed that osteoclasts disappeared in almost all patients, and their histomorphometric parameters changed.[30] Osteoclast surface (Oc.S/BS), eroded surface (ES/BS), and erosion depth (E.De) decreased, whereas osteoblast surface (Ob.S/BS), osteoid volume (OV/BV), osteoid surface (OS/BS), and osteoid thickness (O.Th) increased. In addition, cuboidal osteoblasts were proliferating on the trabecular surface where osteoclasts had been present before the PTX. The rapid decrease in serum PTH levels after PTX appears to suppress bone resorption, as well as cause a transient but marked increase in bone formation.[30,36,37]

Coincidentally, a similar phenomenon was reported not long after the post-marketing of cinacalcet which is a calcimimetic compound used to suppress parathyroid hormone secretion from parathyroid glands in both primary hyperparathyroidism and secondary hyperparathyroidism. A cinacalcet-treated dialysis patient with severe SHPT and increased alkaline phosphatase levels developed hypocalcemia, pronounced hypophosphatemia, and severe diffuse bone pain during treatment.[18,38] An advanced investigation of the relationship between changes in plasma PTH levels and serum phosphorus levels during treatment with cinacalcet found that reduced PTH levels after 13–26 weeks of cinacalcet treatment were associated with corresponding reductions in serum phosphorus. Doses of vitamin D analogs, phosphate binders, and cinacalcet remained unchanged after starting treatment. This study revealed that reductions in PTH that are associated with decreased serum phosphorus during cinacalcet therapy cannot be explained by changes in vitamin D or phosphate binder therapy, and thus may reflect diminished phosphorus release from bone.[17] A 1-year cohort study of 25 hematodialysis patients who had serum PTH levels greater than 300 pg/mL during cinacalcet treatment confirmed that the BMD in the femoral neck increased after 1 year of treatment.[39] These data suggest that cinacalcet treatment might accelerate bone formation by suppressing bone resorption via an inhibitory effect on PTH levels.

In a healthy population, PTH is secreted in a basal mode with superimposed oscillatory bursts every 8–12 minutes.[40] Approximately 30% of circulating PTH is attributable to pulsatile secretion and approximately 70% is due to tonic secretion.[41,42] In secondary hyperparathyroidism due to renal insufficiency, tonic secretion and pulsatile burst mass are also proportionately amplified, and the burst frequency is increased.[41] Continuous exposure of the human skeleton or osteoblasts to PTH or parathyroid hormone related protein (PTHrP) arrests the osteoblast maturation program.[6,43,44,45] *In vitro* experiments lasting 21 days confirmed that continuous PTH treatment blocked osteoblast differentiation. In contrast, transient PTH inhibited initial osteoblast differentiation but ultimately resulted in cultures with more mineralized nodules and enhanced osteoblast differentiation.[46,47] *In vivo* experiments performed with 7 day continuous infusions of hPTH (1–34) and hPTHrP (1–36) in healthy human adult volunteers indicated that bone formation was suppressed by 30–40%.[48] Upon cessation of PTH and PTHrP infusion, bone formation markers (amino-terminal telopeptides of procollagen I and bone-specific alkaline phosphatase) abruptly rebounded. In contrast to continuous PTH administration, intermittent PTH administration stimulated bone formation.[8,49,50]

In SHPT patients, excessive secretion of PTH stimulates bone turnover, accelerates osteoclastic bone resorption, mobilizes bone calcium and results in hypercalcemia and hyperphosphatemia.[14] The higher calcium and phosphorus content in SHPT patient’s serum results from elevated osteoclast activity. In this study, the interference of osteoclast was excluded. Therefore the calcium and phosphorus in MC3T3-E1 cell medium was absorbed by cells to form mineralized nodules. A sustained high dose of bPTH inhibited mineralized nodules formation. And a high-dose bPTH withdrawal removed this inhibition and aroused the formation of mineralized nodules. The higher calcium and phosphorus content in MC3T3-E1 cell medium is supposed to be the decreased absorption by cells.

In summary, our study confirms that withdrawal of bPTH significantly increases AKP activity of MC3T3-E1 cells and the number of mineralized nodules, and causes a rapid decline in calcium and phosphorus levels. The calcium and phosphorus present in the medium were used by the MC3T3-E1 cells to synthesize mineralization material and thus form calcium nodules. According to our clinical data and the results of *in vitro* experiments, we propose that the sudden removal of high levels of PTH leads to an increased influx of calcium and phosphorus into bone after PTX. It should be noted that the results in this study were based on clinical observations and *in vitro* experiments. As there are insurmountable technical difficulties involved in obtaining animals suffering from SHPT with chronic renal failure, we still lack direct evidence to confirm that serum calcium and phosphorus are deposited into bone after PTX. If animal models of severe SHPT associated with chronic renal failure could be established, further experiments will be necessary to prove the hypothesis.
